# Supplementary figures and images for: Negative regulation of angiogenesis and the MAPK pathway may be a shared biological pathway between IS and epilepsy
Source: PLoS One. 2023 Oct 4;18(10):e0286426. doi: 10.1371/journal.pone.0286426 (PMC10550183; doi:10.1371/journal.pone.0286426)

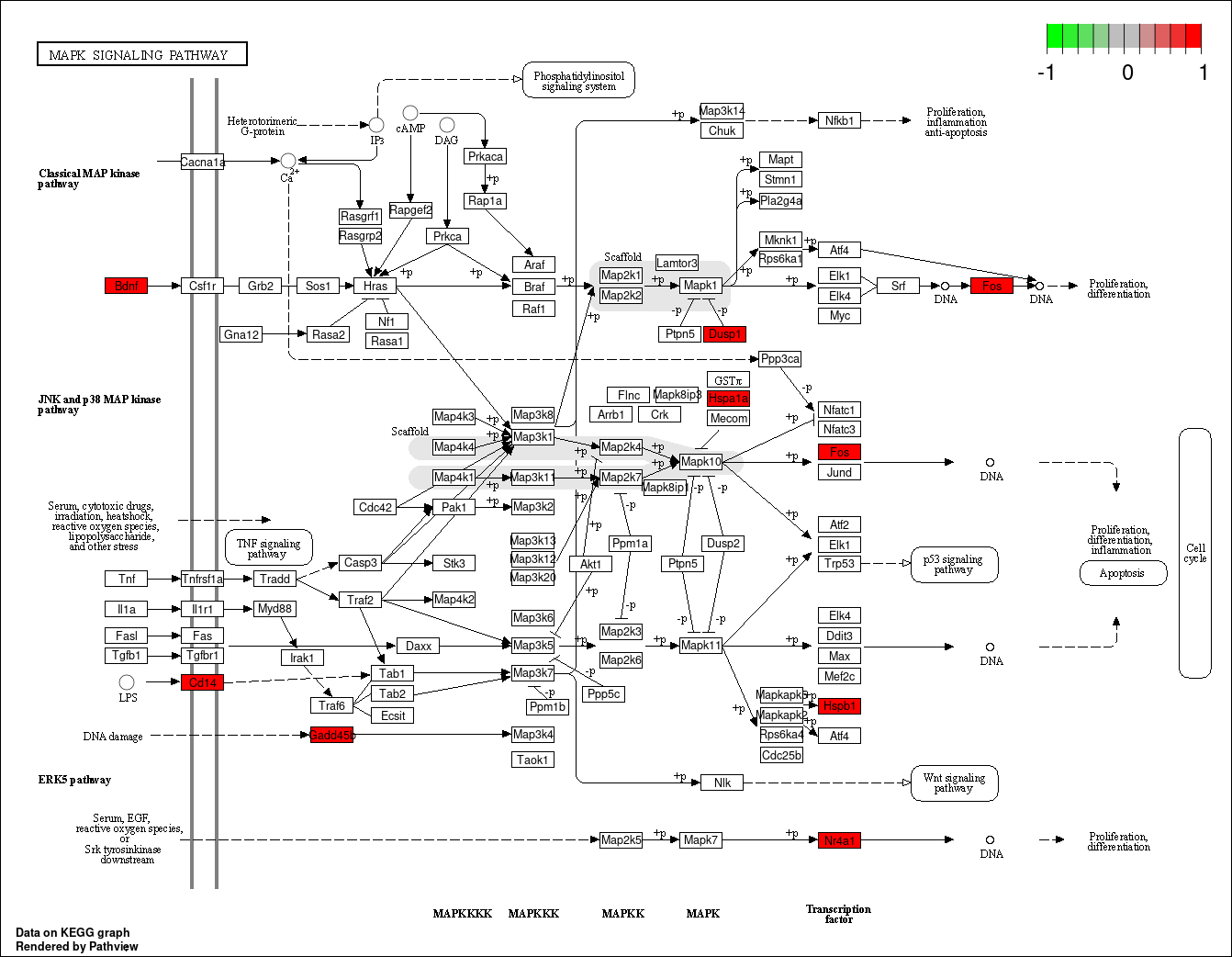

Supplement: S1 Fig — (PNG) [file pone.0286426.s001.png]
