# Supplementary material for: Negative regulation of angiogenesis and the MAPK pathway may be a shared biological pathway between IS and epilepsy
Source: PLoS One. 2023 Oct 4;18(10):e0286426. doi: 10.1371/journal.pone.0286426 (PMC10550183; doi:10.1371/journal.pone.0286426)
Supplement: Attachment — Submitted filename: Response to Reviewers.docx [file pone.0286426.s002.docx]

Dear editor,

On behalf of my co-authors, we thank you very much for giving us an opportunity to revise our manuscript. We appreciate editor and reviewers very much for their positive and constructive comments and suggestions on our manuscript entitled " Negative regulation of angiogenesis and the MAPK pathway may be a shared biological pathway between IS and epilepsy " (Manuscript ID：PONE-D-12-40440).

At the same time, we found some parts of our submission that do not meet the requirements of journal manuscripts, and we have modified them. Thank you for your reminding.

Our reply to the criticisms is as follows:

Reviewer #1: i accept the manuscript titled "Negative regulation of angiogenesis and the MAPK pathway may be a shared biological pathway between IS and epilepsy " as the study concluded that the first comorbidity common between epilepsy and ischemic stroke.

Authors’ response: Thank you for your recognition of our research.

Reviewer #2: 
1. Move the figure legends from the main text to somewhere indicated by the publish policy of Plos One.

Authors’ response: Thank you for your comment. According to the “Submission Guidelines” of Plos One: Figure captions are inserted immediately after the first paragraph in which the figure is cited. Figure files are uploaded separately. Therefore, the figure legends will still appear main text. But thanks for your reminding, we have checked the format of the article and found some formatting problems such as the reference format, and revised this.

2. Authors should consider to change the name of method 2.1 “microarray datasets”, since this part includes bulk and single cell data as well.

Authors’ response: Thank you for your comment. It was a lack of thought, and we apologize for that. We have changed the name of method 2.1 “microarray datasets” to “Datasets acquisition”.Details see page 2, line 71.

3. Detailed parameters used in the software should be disclosed in the method part for reproducibility. For example, in method 2.3, the specific commands used for Metascape and ClueGO, Cytoscape and GSEA should be fully listed. Similar requirements are applied to other packages.

Authors’ response: Thank you for your comment. Our description of the method part is really not detailed enough. We have revised this part according to your comments, focusing on the addition of specific implementation methods including detailed parameters. Details see page 3-4, line 91-116.

4. The session “Data Availability Statement” should be presented somewhere before “Reference”. Authors may have to rearrange some sessions according to the regulation of Plos One.

Authors’ response: Thank you for your comment. This was indeed an oversight on our part, and we adjusted the organizational structure of the article. Details see page 2, line 71.

5. For scRNA-seq analysis in Figure 4, authors should present the cell markers of each cluster annotated in the main figure, in the format of either dotplots or heatmap. This will make the analysis transparent and readable to the readers.

Authors’ response: Thank you for your comment. We have added a bubble chart to show the top 5 marker genes for all 9 cell types, and explained it in the “Results”. Details see page 5, line 145-169, and Fig 4C, D.

6. For scRNA-seq analysis in Figure 4, authors should explain the phenotypes that some cell clusters consist of subpopulations characterized by different sample origin (Sham vs Mcao), especially in the clusters of microglia and ¬endothelial cells. The segregation by conditions indicate that these subsets of cells are condition-specific, and may represent a different cell type/status. Authors should address these issues, and consider to reannotate the clusters accordingly. In addition, the resolution of UMAPs is too low to clearly identify cell labels. High resolution images are required all through the figures.

Authors’ response: Thank you for your comment. As a unified annotation method is required for cells in the MCAO group and the Sham group, we did not use an isolated method to annotate cells in the two groups separately. But we also recognize that the lack of group-based discussion is unconvincing in this section. After thinking, we made some modifications and showed the expression of hub genes based on MCAO and Sham groups respectively, and explained it in the “Results”. We also have some discussion about the subtypes of endothelial cells, as we think they play an important role in both diseases. In addition, we have re-provided TIF files with higher resolution. Details see page 5, line 170-189; page 7-8, line 265-270; Fig 4D, E; Fig 5.

Other changes are marked in red.

Thank you for your comments that help us a lot.
